# Review of "Fractals and Chaos: The Mandelbrot Set and Beyond", by B. Mandelbrot

**DOI:** 10.1186/1475-925X-4-30

**Published:** 2005-05-03

**Authors:** Alberto Diaspro

**Affiliations:** 1 Laboratory for Advanced Microscopy, Bioimaging and Spectroscopy (LAMBS), MicroScoBio Research Center, IFOM, Department of Physics, University of Genoa, Via Dodecaneso, 33 16146 Genoa Italy

Benoit Mandelbrot has produced a comprehensive, well-presented review of essential topics related to Mandelbrot set theory and applications. The last part of the title "The Mandelbrot set and beyond" fully describes its potential allowing the reader to navigate through pictures, hard-to-find early papers and important and effective chapters on the historical background. All chapters are assembled in a way that the overall mix becomes a very well integrated source of know-how and knowledge bringing the readers into the Mandelbrot set world. The spirit of the book is well summarized in a sentence on page 34: "When seeking new insights, I look, look, look, and play with many pictures. (One picture is never enough)." It is certainly true that in the last twenty years, mathematics has changed so deeply that to younger persons some chapter's stories might be simply incredible (p.36), as well, one should admit that after Mandelbrot's sets, initially describing trees, coastlines' shapes or allowing measuring the length of the Britain coast, and after the seminal book on "The Fractal Geometry of Nature" our way of looking at the world changed. Mandelbrot wrote: "Why is geometry often described as 'cold' and 'dry'? One reason lies in its inability to describe the shape of a cloud, a mountain, a coastline or a tree. Clouds are not spheres, mountains are not cones, coastlines are not circles, and bark is not smooth, nor does lightning travel in a straight line". I think our vision of the world, from the atom to the higher length scales, is still changing using those concepts clearly illustrated in the current Mandelbrot's book. Selected notes and papers make this book unique within the several books published on this topic. It is clear the touch of the author under all aspects: a touch of pure genius.

There are five main topics dominating the book, namely: Quadratic iteration and its Mandelbrot set – Quadratic Julia and Mandelbrot sets; Nonquadratic iterations – Nonquadratic rational dynamics; Kleinian groups' limit set – Iterated nonlinear function systems and the fractal limit sets of Kleinian groups; Multifractal invariant measures – Exponentially vanishing multifractal measures; Background and History. Cumulative bibliography is impressive and well done. It is clearly pointed out, following the pathway through the book, how fractal geometry played an important role in offering a quantitative tool in several areas. Circumstances and facts are put together also to bring important lessons for young scientists. The author made a serious and effective effort to realize a book that contains more than history, more than mathematics... it is a sort of ideal book for stimulating new ideas, new concepts, and new discoveries. So far, it is an excellent book also for supporting courses at University, PhD and Post doc level. Moreover, it is indispensable for scientists not only as a lesson of a pathway in science but also as an important source for science of tomorrow. This is a valuable reference source to researchers from these and related areas including bioengineering, biophysics, nanobiosciences and, of course, applied mathematics.

